# Supplementary material for: Child marriage in Canada: A systematic review
Source: PLoS One. 2020 Mar 3;15(3):e0229676. doi: 10.1371/journal.pone.0229676 (PMC7053727; doi:10.1371/journal.pone.0229676)
Supplement: S1 File — (DOCX) [file pone.0229676.s002.docx]

Scopus search terms:

(

TITLE-ABS-KEY((early W/2 marriage*) OR “minimum age for marriage*”)

OR

TITLE-ABS-KEY((adolescen* OR child* OR teen* OR "under age*" OR underage* OR "under 18" OR "below the age of 18" OR young OR youth) W/1 (bride* OR groom* OR husband* OR marriage* OR married OR nuptial* OR wife OR wives))

OR

TITLE-ABS-KEY((adolescen* OR child* OR teen* OR "under age*" OR underage* OR "under 18" OR "below the age of 18" OR young OR youth) W/5 (“arranged marriage*” OR “forced marriage*” OR “servile marriage*” OR “age at first marriage”))

OR

INDEXTERMS(("age at marriage" OR "marriage age" OR (marriage AND “age factors”)) AND (child* OR adolescen* OR youth))

)

AND

TITLE-ABS-KEY(canad* OR quebec OR ontario OR "british columbia" OR winnipeg OR alberta OR saskatchewan OR yukon OR "northwest territories" OR nunavut OR nunavik OR "nova scotia" OR "new brunswick" OR newfoundland OR labrador OR "prince edward island" OR "atlantic provinces" OR maritimes OR Toronto OR Vancouver OR montreal OR Winnipeg OR Ottawa OR Calgary OR Mississauga OR Edmonton OR Brampton OR hamilton)

ProQuest and Canadian Newsstream search terms:

(

NOFT(“child bride*” OR "child marriage*" OR "adolescent marriage*" OR "early marriage*" OR “teen marriage*”)

)

AND

AB,TI,SU(canad* OR quebec OR ontario OR "british columbia" OR winnipeg OR alberta OR saskatchewan OR yukon OR "northwest territories" OR "nova scotia" OR "new brunswick" OR "atlantic provinces" OR maritimes OR newfoundland OR labrador OR nunavut OR nunavik OR "prince edward island" OR Toronto OR Vancouver OR montreal OR Winnipeg OR Ottawa OR Calgary OR Mississauga OR Edmonton OR Brampton OR hamilton)

Google Scholar search terms:

intitle:"child bride"|intitle:"child marriage"|intitle:"adolescent marriage"|intitle:"early marriage"|intitle:"teen marriage" Canada|Canadian
