# Supplementary material for: Child marriage in Canada: A systematic review
Source: PLoS One. 2020 Mar 3;15(3):e0229676. doi: 10.1371/journal.pone.0229676 (PMC7053727; doi:10.1371/journal.pone.0229676)
Supplement: Attachment — Submitted filename: PLOS One Response to Reviewers.docx [file pone.0229676.s003.docx]

Dear Dr. Lawson,

Thank you for the prompt and thorough review of our manuscript. The reviewers’ comments have strengthened the work and we have addressed each of them to the best of our ability. Our detailed responses follow. We have also corrected all of the formatting issues identified in the first round of review.

We wish to note that Alissa Koski will act as corresponding author from this point forward rather than Michele Zaman.

**Responses to Reviewer 1**

Line 35. The word ‘controlled’ here sounds a bit patronizing. Controlled by whom? This could be said in a different way.

Thanks for highlighting this. We agree that the initial phrasing was poor. We have rewritten the sentence to better reflect that married girls report less control over their own fertility, as indicated by their reports of less access to contraception and more unwanted pregnancies.

Lines 46-47, it should be made clear that this is not necessarily a causal relationship, unless you state otherwise.

This is another important point. We have added a statement to this effect following the paragraph on associations between child marriage and health outcomes. See lines 99-117.

Lines 49-53. Same here, which causes which? A growing body of research is suggesting that a lack of affordable and decent quality education in fact leads to early marriage, or that they are co-causal.

We have expanded this discussion to indicate that the relationship likely works in both directions. Strong	evidence from Bangladesh and Uganda indicates that child marriage likely causes girls to leave school,	but in contexts were schooling is inaccessible child marriage may be perceived as an alternative pathway	to adulthood. See lines 119-126.

Lines 177-178: This information about Canadian law should come earlier in the manuscript, at the beginning.

We appreciate the suggestion to restructure the introduction but prefer the style in which it is written.	Even so, we have expanded the last paragraph of the introduction to better explain why the discrepancy	between Canadian domestic law and foreign policy motivated our study.

Line 185: This is one of your conclusions, please make sure it is clearly mentioned there.

We mentioned evidence on age-discrepant marriages in the conclusion section in our first draft, but have	added more detail and rephrased it to make it clearer. See lines 360-363.

Lines 206-209: This is interesting, and perhaps should be highlighted a bit more clearly in your text.

It is interesting, but the authors of the original study were only able to hypothesize about the mechanisms	that may explain these associations. Since this is not an area that either of us have expertise in, we are not	able to	offer further analysis or explanation beyond what the original authors offered, which is outlined	in the text.

Line 209: I am not sure here what you mean by the world ‘ecological’.

We were referring to the fact that the Sen and Ariizumi study uses only provincial-level data, not	individual-level data, and that this limits the degree to which the results can be applied to individuals. We	have omitted the word ‘ecological’ and made this clearer. See line 300.

The ‘Conclusion’ section is for the most part very good and makes a useful contribution to the field, but I would ask the authors to expand on two points within it:

First, the high prevalence of early marriage among teens in Nunavut is quite eye-catching in the data section where it is reported. Do the authors not have any ‘best guesses’ about the reasons behind this? Surely some informed speculation could be ventured here to guide future researchers.

Thank you for pushing us in this direction. We have added additional information on the housing shortage in Nunavut that may contribute to a relatively high prevalence of marriage among teens in the territory. See lines 336-342.

Second, the authors usefully note that aggregation within their data presents difficulties in drawing further conclusions. This could be the springboard for a recommendation for future research: that it should always be disaggregated by age (as well as socio-economic status and so forth). The problem of aggregation affects a broad range of disciplines within the social sciences, so it is always a useful reminder to other researcher to avoid it where possible.

We agree and have added this point to our conclusion. See lines 387-392.

**Response to Reviewer 2**

This is a nicely written paper and clearly makes the case that more research is needed on early marriage in Canada. My one minor comment has to do with the introduction. I would like to see the causal link between early marriage and negative health related outcomes (paragraph 2) questioned or at least a short note suggesting that correlations could be due to selection effects rather than harmful consequences of early marriage per se.

Thank you for making this important point. We have added nuance to our discussion of these	correlations at two points in the introduction: following the paragraph on health-related correlations and	following discussion of lower educational attainment among married girls.
